# Glycosylation Effects on FSH-FSHR Interaction Dynamics: A Case Study of Different FSH Glycoforms by Molecular Dynamics Simulations

**DOI:** 10.1371/journal.pone.0137897

**Published:** 2015-09-24

**Authors:** Biswa Ranjan Meher, Anshuman Dixit, George R. Bousfield, Gerald H. Lushington

**Affiliations:** 1 Bioinformatics Core Facility, University of Kansas, Lawrence, Kansas, United States of America; 2 Institute of Life Sciences, Nalco Square, Bhubaneswar, 751023, Odisha, India; 3 Department of Biological Sciences, Wichita State University, Wichita, Kansas, United States of America; 4 LiS Consulting, Lawrence, Kansas, United States of America; University Hospital of Münster, GERMANY

## Abstract

The gonadotropin known as follicle-stimulating hormone (FSH) plays a key role in regulating reproductive processes. Physiologically active FSH is a glycoprotein that can accommodate glycans on up to four asparagine residues, including two sites in the FSHα subunit that are critical for biochemical function, plus two sites in the β subunit, whose differential glycosylation states appear to correspond to physiologically distinct functions. Some degree of FSHβ hypo-glycosylation seems to confer advantages toward reproductive fertility of child-bearing females. In order to identify possible mechanistic underpinnings for this physiological difference we have pursued computationally intensive molecular dynamics simulations on complexes between the high affinity site of the gonadal FSH receptor (FSHR) and several FSH glycoforms including fully-glycosylated (FSH^24^), hypo-glycosylated (e.g., FSH^15^), and completely deglycosylated FSH (dgFSH). These simulations suggest that deviations in FSH/FSHR binding profile as a function of glycosylation state are modest when FSH is adorned with only small glycans, such as single N-acetylglucosamine residues. However, substantial qualitative differences emerge between FSH^15^ and FSH^24^ when FSH is decorated with a much larger, tetra-antennary glycan. Specifically, the FSHR complex with hypo-glycosylated FSH^15^ is observed to undergo a significant conformational shift after 5–10 ns of simulation, indicating that FSH^15^ has greater conformational flexibility than FSH^24^ which may explain the more favorable FSH^15^ kinetic profile. FSH^15^ also exhibits a stronger binding free energy, due in large part to formation of closer and more persistent salt-bridges with FSHR.

## Introduction

A significant portion of the functional diversity of proteins is derived from their glycosylation states: post-translational modifications that are widely observed in the proteome as a mechanism for fostering the proper folding of some proteins and for thermodynamically stabilizing others. In addition to conformational stabilization, variation in glycosylation states provides a mechanism for controlling protein participation in a wide array of different biochemical processes, playing an especially important role in mediating molecular interactions between cells and their environments [1]. Because of these roles, it is not surprising to discover that glycosylation states of specific proteins have begun to be regarded as sources of phenotypically sensitive biomarkers for a diverse range of health conditions [2]. In most cases, however, the precise kinetic and thermodynamic mechanisms by which glycosylation exacts its biochemical influence are poorly understood [3].

**Fig 1:**
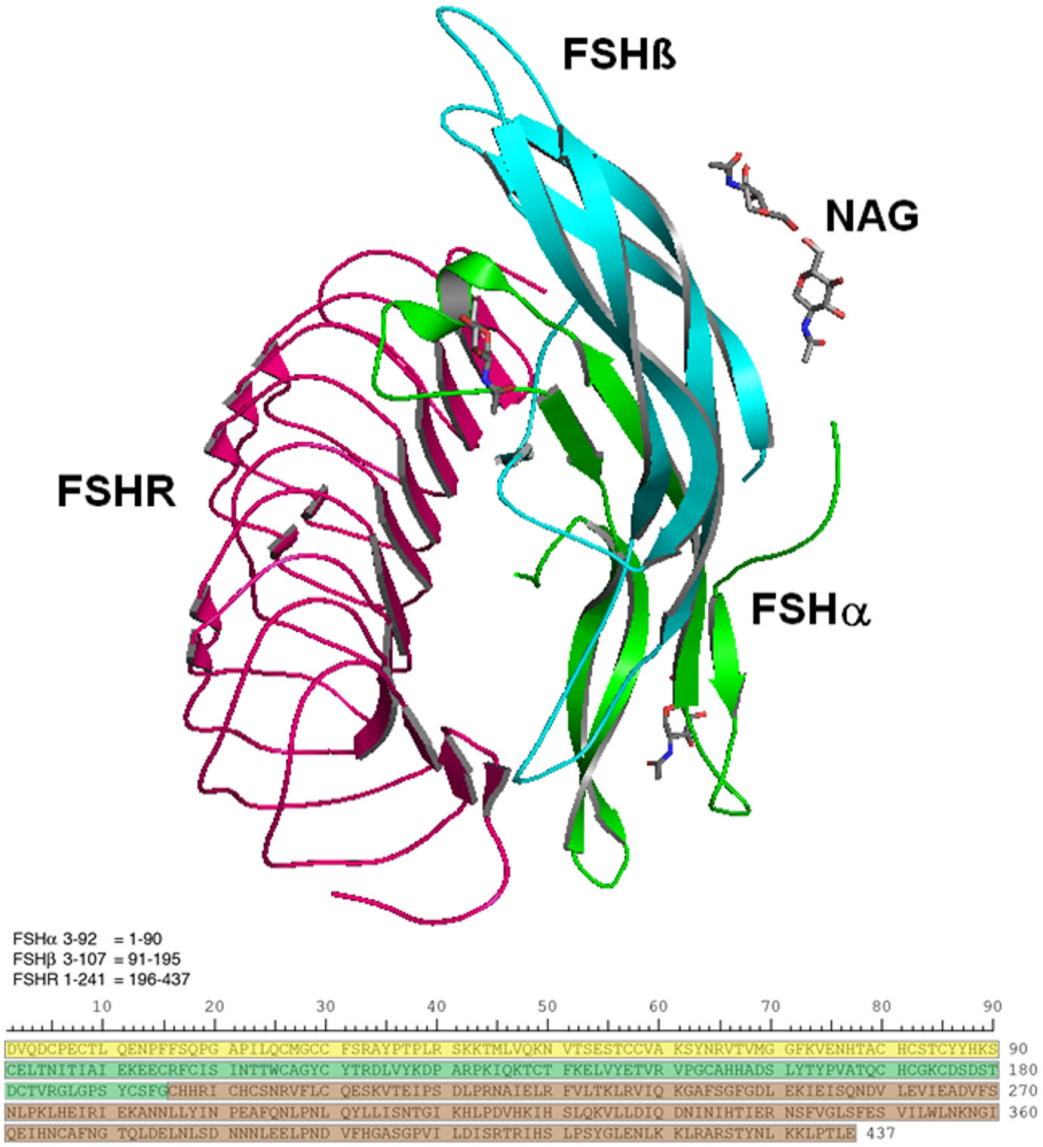
FSH/FSHR quaternary and primary structures examined in this study. (**a**). Structure showing the FSH-FSHR complex with the partial glycosylation of NAG (N-acetylglucosamine). Ribbons colored green and cyan identify FSHα and FSHβ subunits, respectively. The former is glycosylated by NAG at sequence positions 52 and 78, while the latter is glycosylated at positions 7 and 24. NAGs are shown as grey colored stick models. The pink ribbon shows a portion of the FSH receptor extracellular domain that possesses the high affinity FSH binding site. (**b**). The amino acid sequences for FSHα residues 3–92 (yellow), FSHβ 3–107 (green), and FSHR 1–241 (brown) are shown below. In subsequent figures a colored bar will indicate each protein, as the software numbers the residues 1–437.

One particular cellular interaction, whose function and efficacy is known to depend on glycosylation state, is the association of follicle-stimulating hormone (FSH) with its cellular receptor (FSHR). FSH is a gonadotropic hormone responsible for hormonal signaling in the gonads. Heterodimeric in form, FSH shares a highly homologous 92–96 amino acid residue alpha subunit with other glycoprotein hormones, such as luteinizing hormone (LH), thyroid-stimulating hormone (TSH) and chorionic gonadotropin (CG). Each of these hormones exhibits a distinct biochemical function courtesy of their individual beta subunits and from their distinct glycosylation profiles. In the case of FSH, the heterodimer can experience glycosylation at as many as four distinct asparagine residues: FSHα sequence positions 52 and 78 and FSHβ positions 7 and 24. Fig 1 shows the topology and relative orientation of glycosylated FSH in complex with its receptor, FSHR. The critical role of FSH glycosylation state in fostering FSHR activation has been known for some time [4–6], but the more recent discovery of variations in the glycosylation state of human FSHβ, for which hypo-glycosylated variants have been identified [7,8] has fostered a hypothesis that the different isoforms produce key differences in reproductive function [9], and in particular contribute to a decline in female fertility as a function of advancing age [9]. Since fertility treatments have arisen that involve FSHR stimulation via urinary or recombinant FSH preparations (e.g., [10]), it has become particularly important to characterize the dependence of fertility on specific glycosylation states in order to potentially optimize the conditions for fertilization. Another health issue whose phenotypic characterization may also relate to FSH glycosylation state is post-menopausal acceleration of osteoporosis [11].

**Fig 2:**
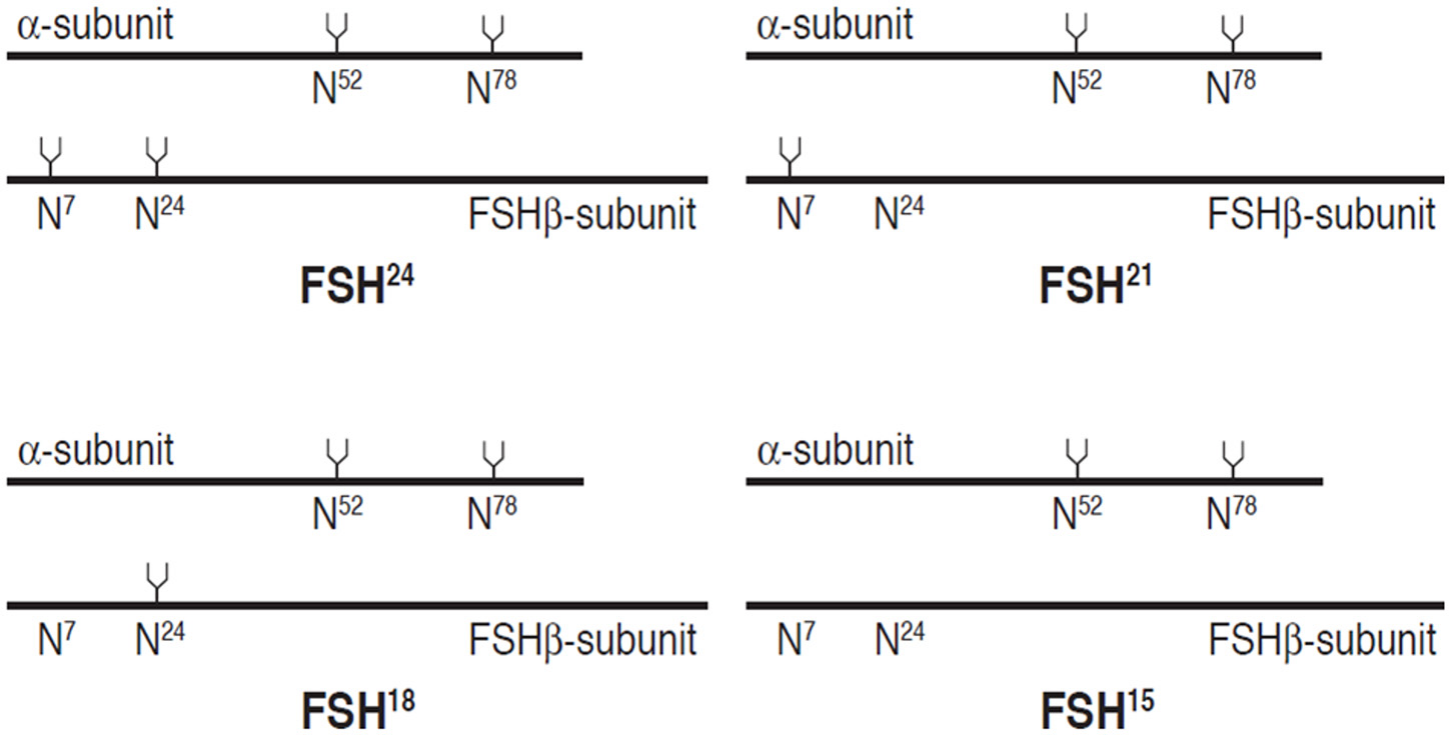
FSH glycoforms result from partial glycosylation of the hFSHβ subunit. The variants are defined by the molecular weights of the FSHβ subunit based on Western blotting experiments. The primary structures are indicated as solid lines, 92 residues for FSHα and 111 for FSHβ. The tuning forks represent N-glycans, when present. The N is the single-letter code for asparagine and the superscript represents each residue’s position in the primary structure.

**Fig 3:**
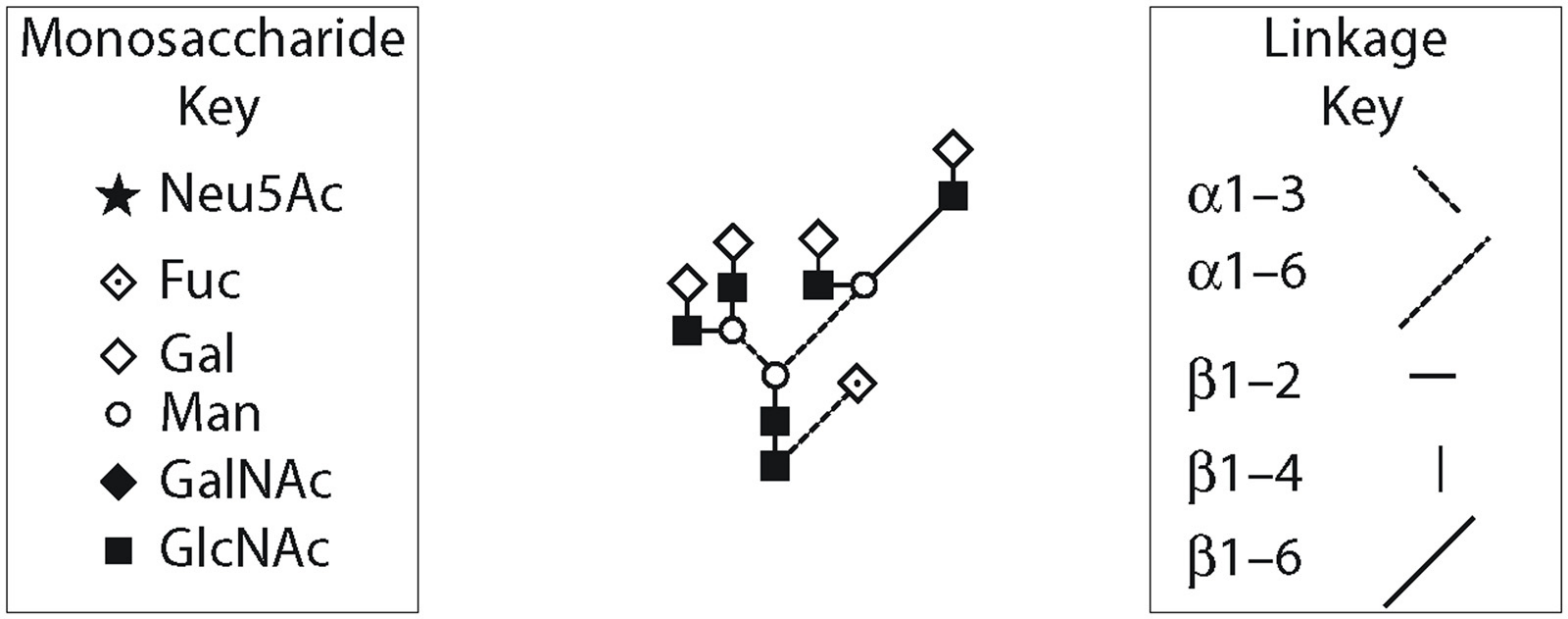
Schematic diagram for the tetra-antennary glycan used in the simulations. The α/β 1–6 linkage line is longer, which indicates the carbon atom is exocyclic. All other linkages involve ring carbons.

Evidence suggests that full glycosylation of FSHα is consistently required for normal biological functions [5,6] but indications are that FSHβ exhibits biological functionality (albeit with varying efficacy toward different cellular targets) when glycosylated at one or both FSHβ N-glycosylation sites [7,12,13]. For the purposes of this study, isoform nomenclature relates to approximate FSHβ molecular weight determined in Western blots, which (with allowances for variation per precise glycan formulation) can be summarized as: FSH^24^ refers to fully glycosylated FSH (i.e., the heterodimer with glycans at both FSHβ asparagine residues; molecular weight approximately 24 KDa), FSH^15^ for fully hypo-glycosylated FSHβ without substitutions at either asparagine (MW ≈ 15 KDa), and FSH^21^ for hypo-glycosylated FSHβ with substitution at only Asn7 (MW ≈ 21 KDa) and FSH^18^ for hypo-glycosylated FSH with substitution at only Asn24 (MW ≈ 18 KDa). The FSHα subunit always possesses both N-glycans and migrates as a single band following SDS-PAGE, albeit with varying mobilities depending on the glycan populations attached. These FSH glycoform variants are summarized in Fig 2. The composition and structure of the tetra-antennary glycan used in the simulations is shown in schematic form in Fig 3 using the Oxford Glycobiology Institute system [14,15], with one modification in order to provide the readers a better understanding of the structure—the α/β 1–6 linkage line is made longer indicating the carbon atom is exocyclic. All other linkages involve ring carbons.

While FSH^24^ appears to predominate in men and post-menopausal women, FSH^21^ exhibits elevated abundance in younger females of normal child-bearing age, which has led to suggestions that FSH hypo-glycosylation may play a preferential role in efficient stimulation of ovarian follicle development, and thus serve as a regulator of reproductive fertility [13]. Interestingly, crystallographic evidence clearly suggests that the FSH / FSHR association produces a complex which primarily orients FSHβ glycans away from the receptor [16,17]. Thus, any enhanced biochemical signaling effected by the hypo-glycosylated isoform is likely due not to alleviating glycan/receptor steric clash, but instead, is more likely related either to a change in FSH conformation (to make it more thermodynamically suitable for FSHR binding) or FSH flexibility (to render the ligand more kinetically facile for coupling). The relative prospects for either scenario may be conveyed by kinetic observations: a relatively rapid FSH/FSHR association accompanied by similar complex persistence behavior would tend to suggest a kinetically delimited mechanism (i.e., more likely governed by enhanced flexibility of hypo-glycosylated FSH), while similar associative kinetics accompanied by a significant discrepancy in complex persistence would more reflect thermodynamic factors. Preliminary kinetic evidence suggests a substantial difference in the kinetics associated with the onset of FSH/FSHR complexation [18].

An ideal basis for attempting to understand the glycoform-dependence of FSH/FHSR kinetics and associative thermodynamically regulated processes is with structural biological characterization of the specific participants in these processes; in particular FSH glycoforms and the various other proteins with which they may meaningfully interact. Unfortunately, FSH has proven challenging to structurally characterize; at the time that this study was completed there were two relevant crystal structures at our disposal (of the FSH heterodimer alone [19] and in complex with the binding site portion of FSHR [16]; with the complex with the entire FSHR extracellular domain having been reported quite recently [20]). The practical challenges faced in obtaining these structures have made it unlikely that a high throughput crystallographic protocol will avail itself for the purpose of resolving a potentially substantial number of distinct glycoforms corresponding to differential substitution on the β subunit and population by a variety of different glycans at the four glycosylation sites on the FSH heterodimer. A more practical solution would be to apply the existing structural knowledge of FSH and its receptor complex toward molecular dynamics (MD) simulations of relevant glycoforms, thus achieving a fairly rigorous assessment of structural, energetic and kinetic dependencies arising from varied glycosylation state. The work reported herein is intended to serve as a proof-of-principle study, with a specific goal of using simulations to probe whether the superior binding kinetics of hypo-glycosylated FSH are related to greater structural FSH flexibility, greater FSH binding free energy, or perhaps both. The study is also intended as a means for laying a foundation for deconvoluting the associative mechanism and thus potentially providing a vehicle for devising FSH glycoforms with specialized FSHR binding attributes. To this end we have pursued MD simulations that contrast the behavior of hypo-glycosylated FSH (FSH^15^) versus FSH^24^ via adornment by two different model glycans. These two glycans include one of the smallest (a single N-acetylglucosamine (NAG) residue, which reflects chemically [4] and endoglycosidase F [16], [17] deglycosylated FSH) and one of the largest (a tetra-antennary glycan (TAG)) of relevance to FSH studies. For both glycan models, simulations were pursued with glycosylation at either two or four sites on the FSH. In addition to simulations on FSH^15^ and FSH^24^ with different glycans, the dynamic behavior of fully deglycosylated FSH (which we will refer to as dgFSH) was studied. In all of these cases, the FSH behavior was examined in complex with that portion of the FSHR receptor crystallographically resolved by Fan and Hendrickson [16], as these studies preceded the more recent structure reported by Jiang *et al*. [17].

## Materials and Methods

### 2.1 System Setups

The crystal structure of the FSH-FSHR complex (PDB code 1XWD; 2.92 Å resolution) [16] with a single domain conformation was used as the starting structure for the MD simulations. A total of five distinct simulations were carried out for this study, referenced as follows according to glycosylation state and specific model glycan attached: (**1**) dgFSH/FSHR; (**2**) FSH^15^(NAG)/FSHR; (**3**) FSH^24^(NAG)/FSHR; (**4**) FSH^15^(TAG)/FSHR; and (**5**) FSH^24^(TAG)/FSHR.

As the crystal structure is populated with NAG glycans at all four asparagine residues resulting from endoglycosidase F-treatment (structure 3 above), we used this as the FSH^24^(NAG) model and then constructed preliminary structures for the deglycosylated and hypo-glycosylated NAG proteins (structures 1, 2 above) by manually deleting the relevant glycan residues using SYBYL [21]. The TAG glycan model was constructed using the web-based Carbohydrate Builder provided by Dr. R.J. Woods, University of Georgia [22] and was incorporated onto the FSH framework using SYBYL. The *tleap* module of AMBER10 software [23] was used to protonate each of the protein-ligand complexes and to prepare the associated topology and coordinate files, specifying ff99SB [24] force field parameters for the proteins and the Glycam06 force field parameters [25] for glycans. The system was solvated with the TIP4P [26,27] water models in the periodic box of size 89.2 x 84.8 x 96.3 Å^3^ containing more than 17,000 water molecules. Explicit aqueous solvation boxes were constructed for each complex with buffering distance of 10 Å; assuming normal charge states of ionizable groups corresponding to pH = 7.0, sodium (Na^+^) ions were added to achieve charge neutrality in a manner that mimics a biological environment.

For this study, we implemented a rigorous and systematic protocol for alleviating non-optimal atomic contacts arising from the automated protonation, solvation and charge neutralization. This entailed both molecular mechanics and MD refinement courtesy of NAMD, a highly scalable modeling software [28]. NAMD is suitable for efficient parallel simulations executed over a large number of computational nodes; most of our simulations were executed over 32 processor cores in the present study.

The first step in our structural refinement protocol involved initial molecular mechanics minimization for 20000 steps keeping protein backbone fixed, followed by 20000 steps of minimization with no constraints (i.e., completely free relaxation). Analysis of the atomic force distribution on the resulting structure suggested an effective removal of unphysical contacts that might have otherwise imparted unrealistic forces onto subsequent MD simulations.

For the various MD steps involved in structure refinement and in the subsequent analytical simulations, an integration time step of 2.0 fs was used. Non-bonded van der Waals interactions were modeled using a switching function at 10Å and extending out to a distance of 12Å. The particle mesh ewald algorithm, PME [29] implemented in NAMD was used to handle long range electrostatic forces. For early steps in which chemical bonds to hydrogen atoms were fixed at their equilibrium covalent distances, the SHAKE algorithm [30] was applied, whereas heavy atom constraints were affected via the SETTLE [31] protocol.

### 2.2 Molecular Dynamics Simulations

Each complex was adapted for MD simulation by incremental heating followed by isobaric equilibration. Specifically, the system temperature was gradually increased in increments of 20K until reaching a simulation temperature of 300K, performing 15000 simulation steps (30 ps) equilibration at each temperature increment, with hydrogen covalent bond distance fixed and with application of a mild restraint of 10 Kcal mol^-1^ Å^-2^ on amino acid α-carbons (C_α_) in order to preserve the basic protein folds. Thereafter the system was permitted a further equilibration of 150,000 steps (300ps) at 310K (constant volume), followed by 150,000 steps (300ps) at 310K at constant pressure using a Langevin piston to achieve a relatively stable cell size. All restraints were then removed as each system was equilibrated for 500,000 steps (1 ns) at constant volume and 500,000 steps (1 ns) at constant pressure. Finally, a constant pressure simulation was performed for each system run on the equilibrated structure for 40 ns at 1 bar pressure and 310K.

For characterization purposes, the analytical trajectories for each system were interrogated in order to describe the root mean squared cumulative deviation of backbone atom positions (RMSD) relative to the equilibrated structure, and the root mean squared fluctuations in backbone atomic positions (RMSF). Persistence of key salt bridges stabilizing the FSH/FSHR interface were also tracked by sampling, after every 1000^th^ time step, the N-O distance between charged atoms in arginine and lysine (N / cationic) versus aspartate and glutamate (O / anionic) side-chains within the interface region.

### 2.3 MM-GBSA Calculations

Finally, in order to quantify the relative FSH/FSHR binding free energy in each system, the structure of each system (as equilibrated per prior description via NAMD) was subjected to MM-GBSA (Molecular Mechanics Generalized Born Surface Area) based calculations [32] as implemented in the AMBER10 package [23]. The force field and charges used for these calculations were identical to those specified for the previous NAMD simulations. Binding energies were quantified via snapshots collected 1 ns apart for the entire simulation. For each complex, a total number of 40 snapshots were taken. All other parameters associated with the MM-GBSA calculations were left at standard defaults, with the exception that changes in conformational entropy were deemed to be negligible (by virtue of the very modest RMSD and RMSF profiles evident from the earlier simulations) and were omitted from consideration.

The MM-GBSA method in general can be summarized as:

$$\Delta G_{bind}\;or\;GBTOT\;=\;\Delta E_{MM}\;+\;\Delta G_{sol}\;-\;T\Delta S$$ (1)

where Δ*G*_bind_ is the binding free energy in solution consisting of the molecular mechanics free energy (Δ*E*_MM_), the solvation free energy (ΔG_sol_) and the conformational entropy effect due to binding (-TΔS). However, in the present study entropy calculations were deemed negligible and omitted from being consideration as described above.

Hence, the MM-GBSA method of calculations followed in this study can be summarized as:

$$\Delta G_{bind}\;or\;GBTOT\;=\;\Delta E_{MM}\;+\;\Delta G_{sol}$$ (2)

Δ*E*_MM_ can be expressed as:

$$\Delta E_{MM}\;=\;\Delta E_{vdw}\;+\;\Delta E_{ele}$$ (3)

where Δ*E*_vdw_ and Δ*E*_ele_ correspond to the van der Waals and electrostatic interactions in the gas phase, respectively. The solvation free energy (Δ*G*_sol_ or GBSOL) is further divided into two components:

$$\Delta G_{sol}\;or\;GBSOL\;=\;\Delta G_{pol}\;+\;\Delta G_{nonpol}$$ (4)

where Δ*G*_pol_ and Δ*G*_nonpol_ are the polar and non-polar contributions to the solvation free energy, respectively. The Δ*G*_sol_ is calculated with the PBSA module of the AMBER suite of programs. In our calculation, the dielectric constant is set to 1.0 inside the solute and 80.0 for the solvent. The nonpolar contribution of the solvation free energy is calculated as a function of the solvent-accessible surface area (SASA), as follows:

$$\Delta G_{nonpol}\;=\;\gamma (SASA)+\;\beta$$ (5)

where the values of empirical constants γ and β were set to 0.00542 kcal/(molÅ^2^) and 0.92 kcal/mol, respectively. Graphic visualization and presentation of protein structures were performed using PyMol software [www.pymol.org].

## Results and Discussions

### 3.1 RMSD Analysis

**Fig 4:**
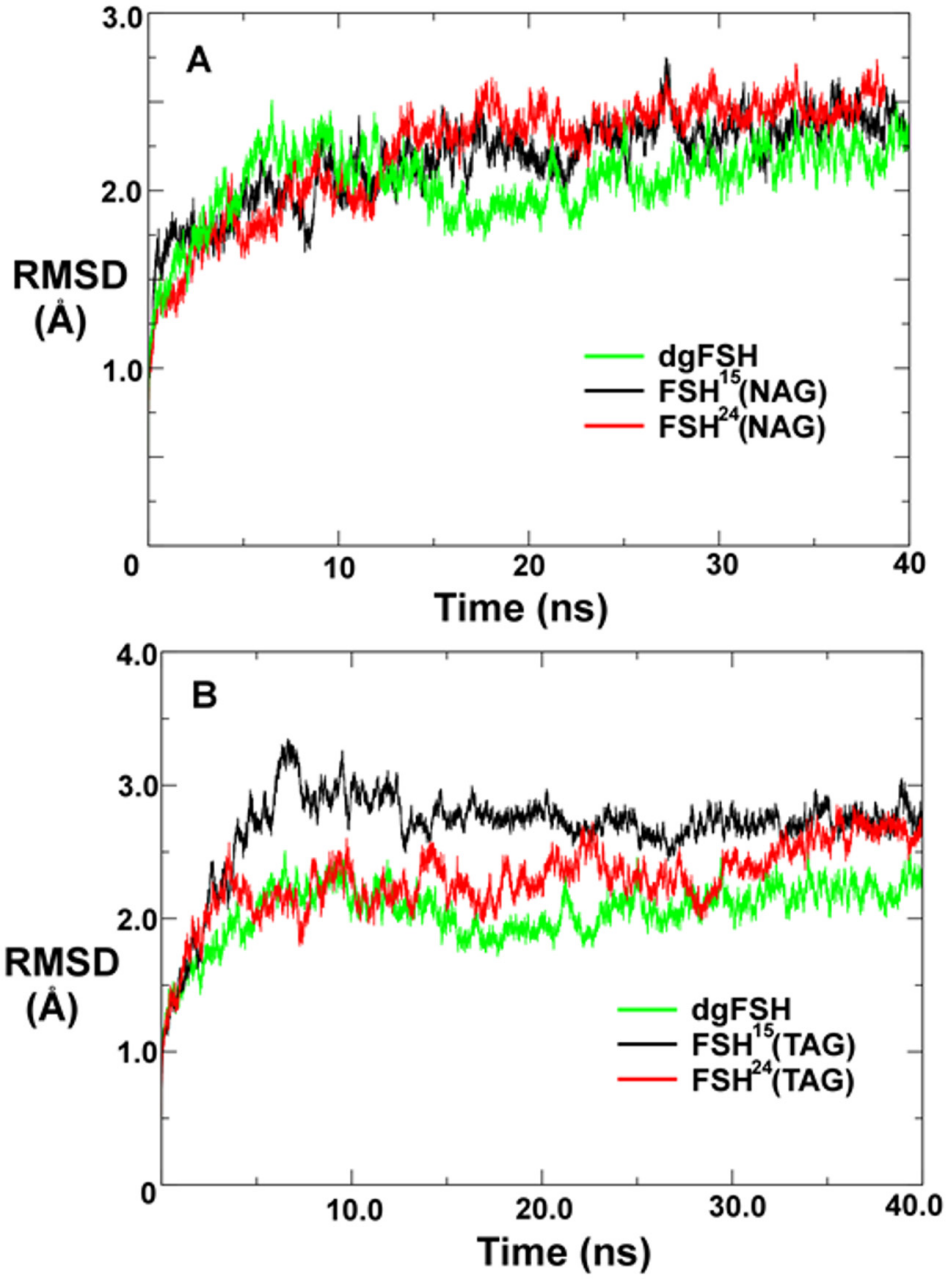
Root mean-squared positional deviation among FSH protein backbone atom positions during molecular dynamics simulations computed relative to the original X-ray crystallographic coordinates. **A**. Comparison of FSH glycoforms decorated with single NAG residues. **B**. Comparison of FSH glycoforms decorated with TAG oligosaccharides.

RMSD analysis for the different complexes is reported in Fig 4. In all cases, the structure tends to undergo a minor shift (RMSD > 2.0 Å) within the first 5 ns of simulation, followed by adherence to a relatively stable conformation thereafter. This is a fairly modest level of structural shift that could primarily be ascribed to the difference in simulation temperature (310K) relative to the conditions under which crystal characterization typically is performed (typically less than 100K depending on the precise technique; in this case the precise temperature is not reported for the originating crystal structure [13]). The only other significant impetus for structural relaxation is if a substantial modification is made to the structure that appreciably disrupts the equilibrated balance of forces present in the crystallized structure. Since the originating crystal structure is glycosylated on all four relevant asparagine residues, and since the crystallized glycan on each site is a single NAG residue, it would be normal to expect no substantial structural relaxation of the simulated FSH^24^(NAG) system. It can thus be considered to be a baseline reference by which to assess structural relaxation by the other systems. We thus find from Fig 4A that none of the other NAG-substituted FSH variants appear to relax in a manner that is qualitatively much different from FSH^24^(NAG). Furthermore, the introduction of balanced modifications such as removing all four glycans or substituting all four NAG residues with the much larger TAG appendage, seem to have little effect on the overall structural stability of the system. However, the unbalanced FSH^15^(TAG) structure behaves in a markedly distinct fashion by exhibiting a sharp structural shift between 5–10 ns into the simulation (Fig 4B).

**Fig 5:**
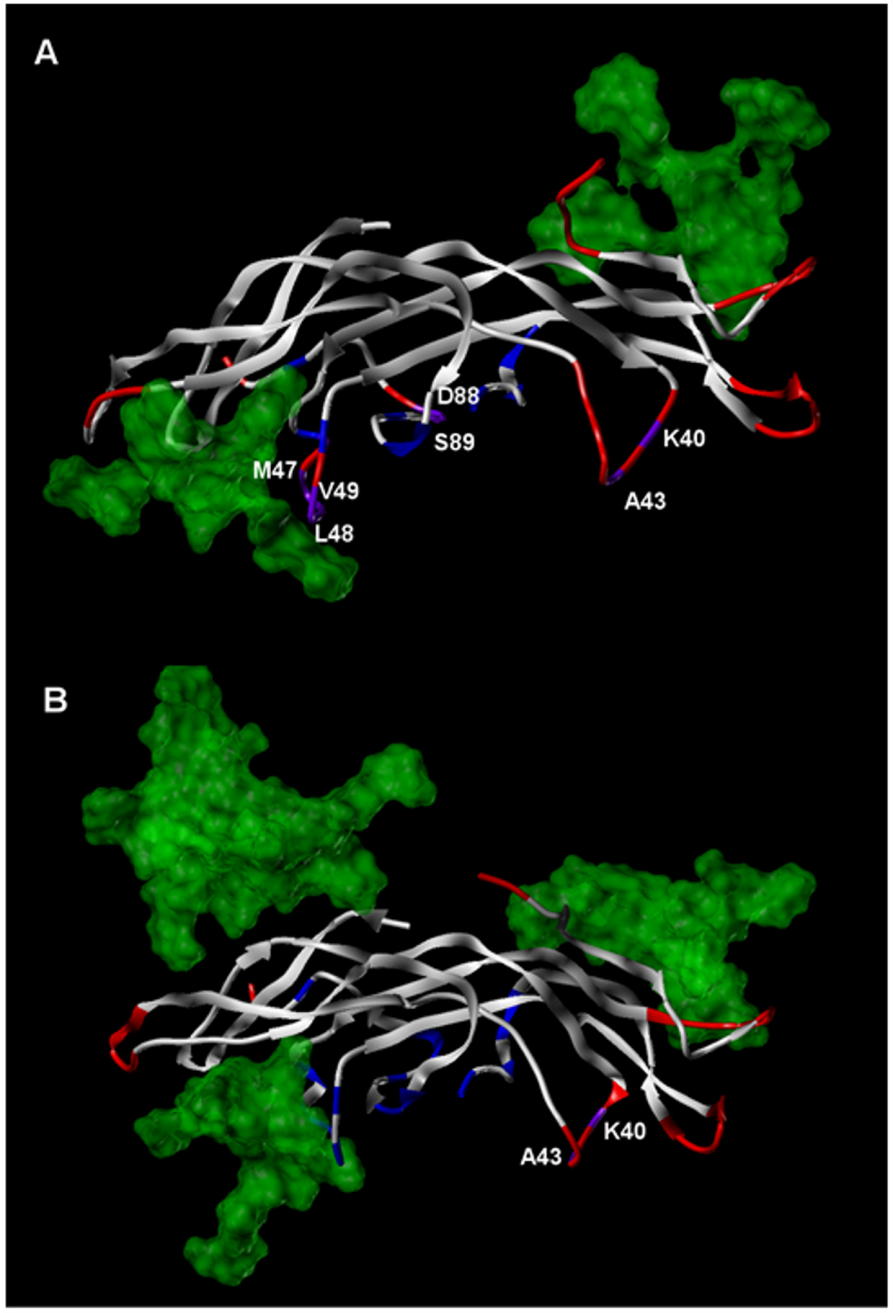
Comparison of FSH dynamic structural features at the FSH/FSHR interface for FSH(TAG) glycoforms. A. FSH^15^(TAG) and B. FSH^24^(TAG). The protein portion of FSH is rendered in ribbon form, while the spatial extent of TAG glycan residues is shown via transparent green features. FSH ribbons are colored as follows: blue = FSH residues with FSHR contact surfaces; red = FSH residues with backbone RMSD shifts of greater than 1.5 Å; purple = FSH residues with FSHR contact surfaces and backbone RMSD shifts of greater than 1.5 Å.

It can be noted from Fig 4B that over the course of a full 40 ns simulation, the overall RMSD of FSH^24^(TAG) and dgFSH eventually attain a level comparable to FSH^15^(TAG), however, the distribution of positional deviation in FSH^15^(TAG) is not equivalent to FSH^24^(TAG). As shown in Fig 5A and 5B, we find that the precise glycosylation state has a substantial effect in determining which specific residues exhibit the greatest shifts relative to the original crystal structure. Regardless of glycosylation state, the percentage of residues that exhibit backbone RMSD shifts of greater than 1.5 Å from the equilibrated positions is relatively small. Nearly all of the overall variation that is observed in each structure occurs on a solvent exposed surface, and within that subset the predominant fraction of total variation is localized mostly either in flexible loops and chain termini. Relatively few high mobility residues are found in close proximity to the FSH/FSHR interface where they would have the greatest influence on FSH binding kinetics. In the case of residues located at or near the FSH^24^(TAG)/FSHR interface, only Lys40 and Ala43 (both in FSHβ) exhibit positional shifts of greater than 1.5 Å, while for the FSH^15^(TAG)/FSHR interface that list includes Lys40, Ala43, Asp88, and Ser89 in FSHβ plus Met47, Leu48 and Val49 in FSHα.

### 3.2 Comparing the proteins by RMSF

We also analyzed protein flexibility variations computed from the root mean square fluctuation (RMSF) of the Cα atoms of residues. To facilitate the analysis, the average protein flexibility profiles were mapped onto the respective conformational states of different glycosylated and non-glycosylated FSH subunits only (dgFSH, FSH^15^ and FSH^24^) and FSH-FSHR interacting complexes (dgFSH-FSHR, FSH^15^-FSHR, FSH^15^(NAG)-FSHR, FSH^15^-FSHR and FSH^24^(TAG)-FSHR) using a color-coded sliding scheme. The color-coded sliding scheme corresponds to the following ranges of protein flexibility values: red (highly flexible with +5.00 Å values), brown (+4.00 Å values), yellow (+3.00 Å), green (+2.00 Å), cyan (+1.00 Å) and blue (the least flexible). In agreement with the structural factors, the regions of larger thermal fluctuations and the increased conformational flexibility corresponded to β-hairpin loops of both the FSH monomers, termini of both the FSH and FSHR.

#### 3.2.1 RMSF of dgFSH, FSH^15^ and FSH^24^

**Fig 6:**
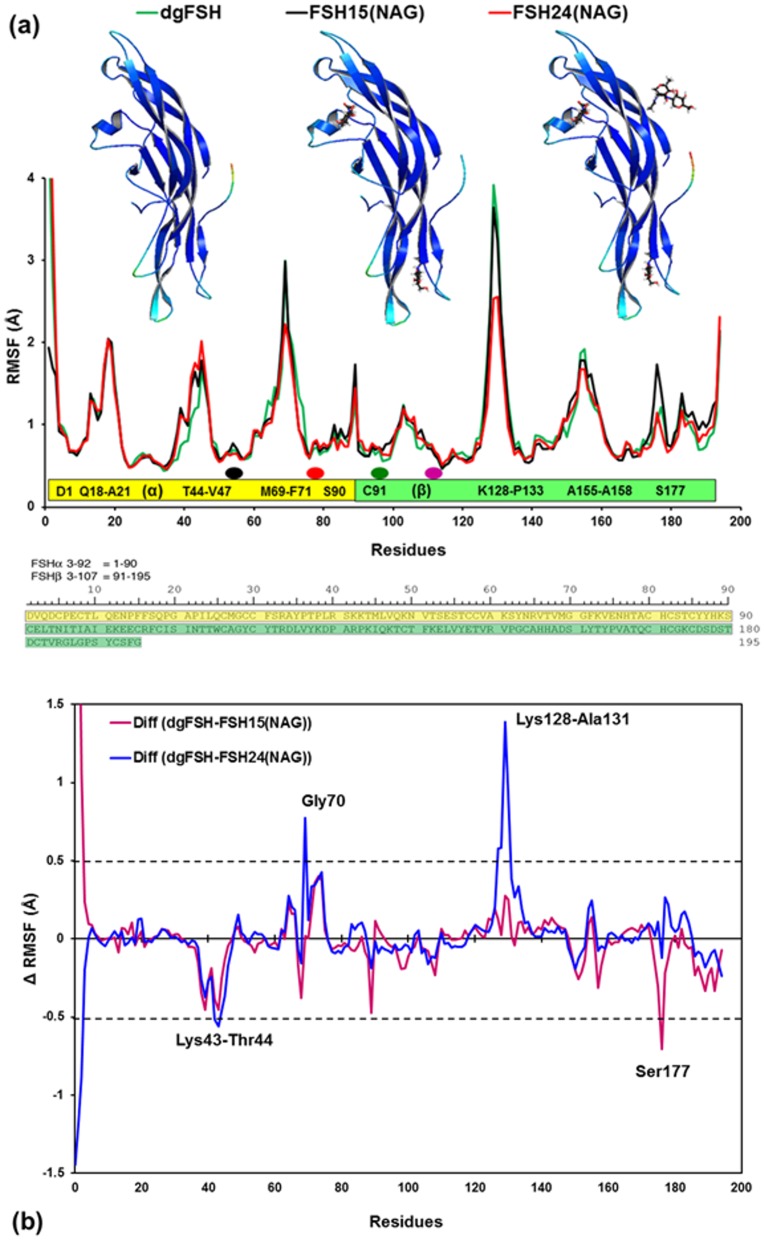
**(a)** Plot showing RMSF values of Cα atoms from MD simulations of dgFSH (in green), FSH^15^(NAG) (in black), and FSH^24^(NAG) (in red). Residues associated with the RMSF, showing each subunit as a bar (α-subunit: yellow bar and β-subunit: green bar) and single-letter code sequences with residue numbers for the regions where RMSF changes reasonably. Oval dots over the bar are shown for the potential N-glycosylation sites (Black & Orange dots for Asn52 & Asn78 on FSHα and Green & Purple dots for Asn7 & Asn24 on FSHβ). Residue sequences with reasonable RMSF changes of at least >1.0 Å are labeled inside the bars in each subunit. Ribbon models: Color-coded mapping of the averaged protein flexibility profiles (RMSF values) from MD simulations of the dgFSH, FSH^15^(NAG) and FSH^24^(NAG) (from left to right). The color-coded sliding scheme corresponds to the following ranges of protein flexibility values: red (highly flexible with +5.00 Å values), brown (+4.00 Å values), yellow (+3.00 Å), green (+2.00 Å), cyan (+1.00 Å) and blue (the least flexible). The amino acid sequences for FSHα residues 3–92 (yellow), and FSHβ 3–107 (green) are shown in the lower panel of the figure. **(b)** Difference of RMSF values for FSH^15^(TAG) and FSH^24^(TAG) from dgFSH. The specific residues with absolute difference larger than 0.50 Å (dashed lines) are labeled.

The RMSF for the three FSH glycoforms (dgFSH, FSH^15^ and FSH^24^) is shown in Fig 6a. The average RMSF values per-residue for dgFSH, FSH^15^ and FSH^24^ are 1.014 Å, 0.999 Å and 1.018 Å, respectively. It suggests that the global changes in RMSF for these glycoforms are neither extensive nor global. Rather, there exist specific local changes, which occur mostly in the β hairpin-loops, P-loops and termini of both FSH subunits. Differences in the RMSF values of the protein moieties for FSH^15^ and FSH^24^ from those of dgFSH are shown in Fig 6b. Residues with absolute difference more than 0.50 Å (dashed lines) were considered as highly fluctuating residues and were labeled. There are significant differences for the following residues: Lys43—Thr44 (FSHα Helix region), Gly70 (FSHβ hairpin loop), Lys128—Ala131 (FSHβ P-loop), and Ser177.

#### 3.2.2 RMSF of dgFSH, FSH^15^(NAG)-FSHR and FSH^24^(NAG)-FSHR

**Fig 7:**
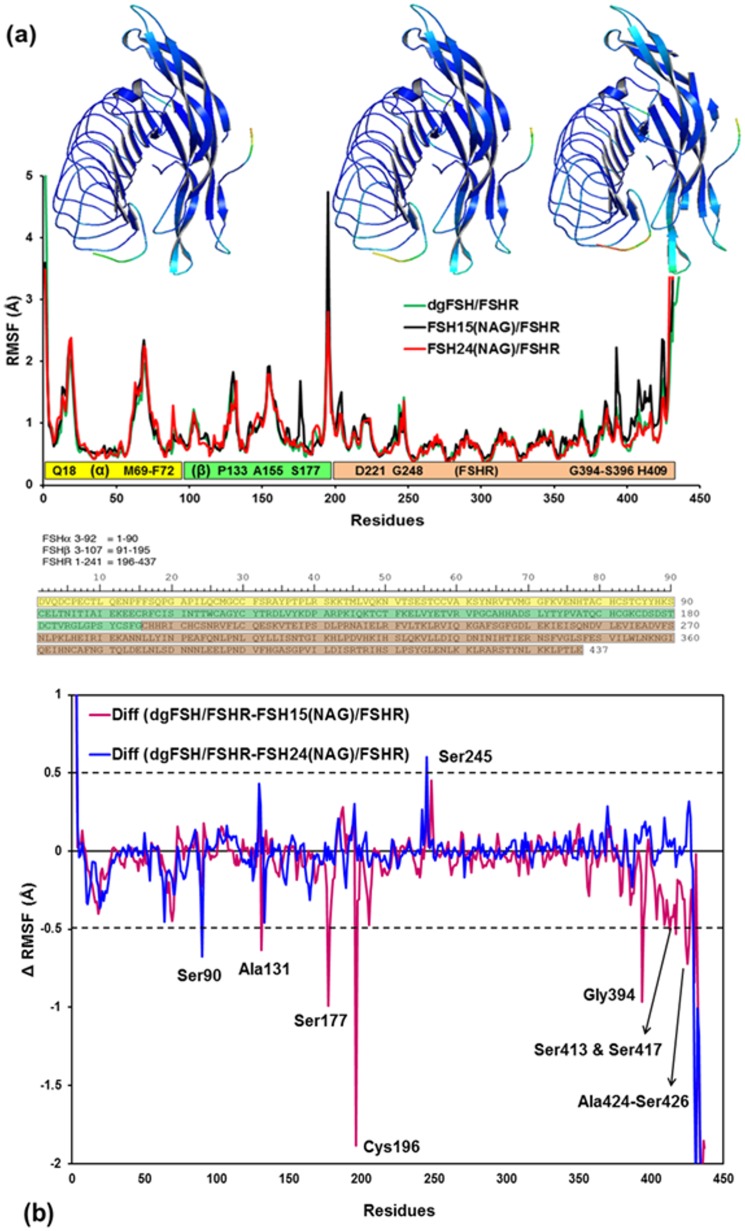
**(a)** Plot showing RMSF values of Cα atoms from MD simulations of dgFSH / FSH^24^(TAG) (in green), FSH^15^(TAG) (in black), and FSH^24^(TAG) (in red). Residues associated with the RMSF, showing each subunit as a bar (α-subunit: yellow bar, β-subunit: green bar and FSHR: light-orange bar) and single-letter code sequences with residue numbers for the regions where RMSF changes reasonably. Residue sequences with reasonable RMSF changes of at least >1.0 Å are labeled inside the bars in each subunit. Ribbon models: Color-coded mapping of the averaged protein flexibility profiles (RMSF values) from MD simulations of the dgFSH, FSH^15^(TAG) and FSH^24^(TAG) FSH-FSHR complexes (from left to right). The color-coded sliding scheme is the same as was adopted for Fig 6a. The amino acid sequences for FSHα residues 3–92 (yellow), FSHβ 3–107 (green), and FSHR 1–241 (brown) are shown below. **(b)** Difference of RMSF values for FSH^15^(TAG) and FSH^24^(TAG)from dgFSH. The residues with absolute differences greater than 0.50 Å (dashed lines) are labeled.

RMSF for each of the three FSH glycoforms complexed to the FSHR (dgFSH, FSH^15^(NAG) and FSH^24^(NAG)) is shown in Fig 7a. The average RMSF values per-residue for dgFSH, FSH^15^(NAG) and FSH^24^(NAG) are 0.850 Å, 0.934 Å and 0.888 Å, respectively. Differences in the RMSF values of the protein for FSH^15^(NAG) and FSH^24^(NAG) from dgFSH are shown in Fig 7b. Considerable differences exist for the following residues: Ser90, Ala131, Ser177, Ser245, Gly394, Ser413, Ser417, Ala424 and Ser426.

#### 3.2.3 RMSF of dgFSH, FSH^15^(TAG)-FSHR and FSH^24^(TAG)-FSHR

**Fig 8:**
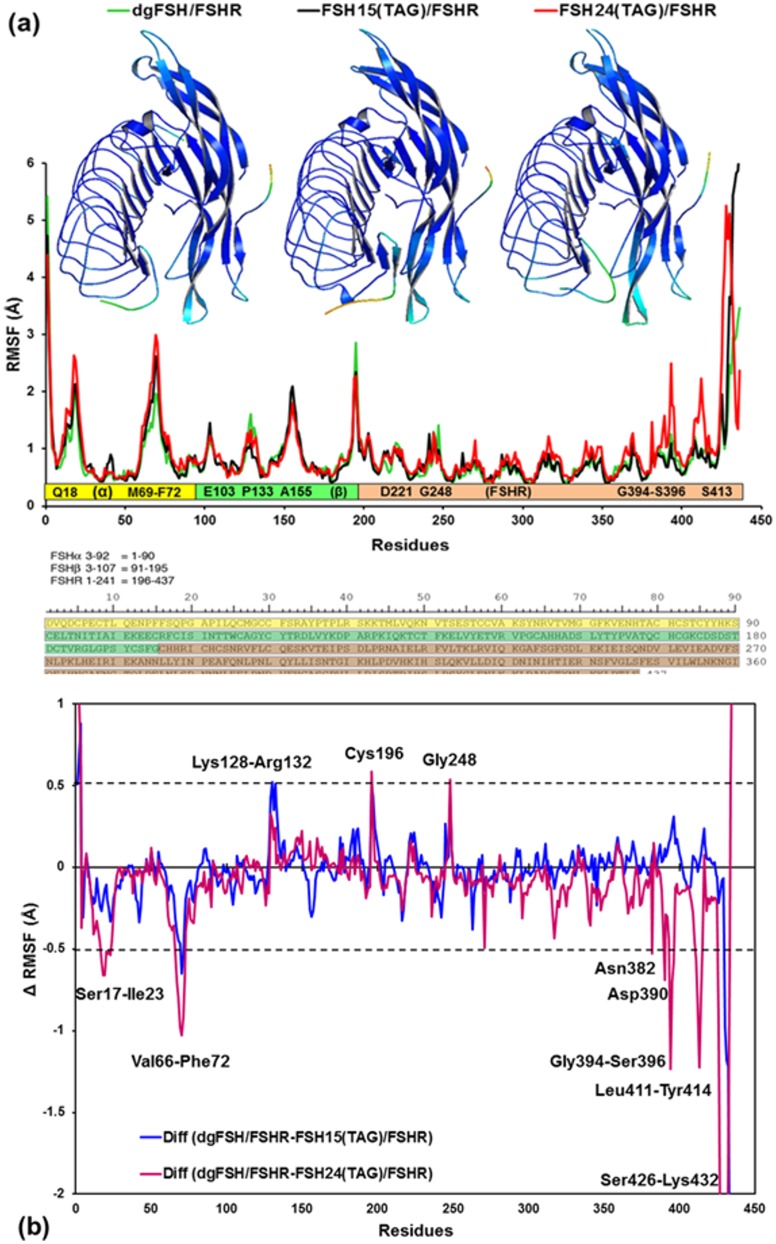
**(a)** Plot showing RMSF values of Cα atoms from MD simulations of dgFSH / FSH^24^(TAG) (in green), FSH^15^(TAG) (in black), and FSH^24^(TAG) (in red). Residues associated with the RMSF, showing each subunit as a bar (α-subunit: yellow bar, β-subunit: green bar and FSHR: light-orange bar) and single-letter code sequences with residue numbers for the regions where RMSF changes reasonably. Residue sequences with reasonable RMSF changes of at least >1.0 Å are labeled inside the bars in each subunit. Ribbon models: Color-coded mapping of the averaged protein flexibility profiles (RMSF values) from MD simulations of the dgFSH, FSH^15^(TAG) and FSH^24^(TAG) FSH-FSHR complexes (from left to right). The color-coded sliding scheme is the same as was adopted for Fig 6a. The amino acid sequences for FSHα residues 3–92 (yellow), FSHβ 3–107 (green), and FSHR 1–241 (brown) are shown below. **(b)** Difference of RMSF values for FSH^15^(TAG) and FSH^24^(TAG) from dgFSH. The residues with absolute difference larger than 0.50 Å are labeled by two cutoff dashed black lines.

RMSF for the three FSH glycoforms complexed to the FSHR (dgFSH, FSH^15^(TAG) and FSH^24^(TAG)) is shown in Fig 8a. The average RMSF values per-residue for dgFSH, FSH^15^(TAG) and FSH^24^(TAG)are 0.85 Å, 0.90 Å and 0.98 Å, respectively. It suggests that global differences in RMSF for these glycoforms are observed, and conformational effects vary as a function of the numbers of TAG bonded to the protein. Moreover, for the FSH-FSHR complex, the average residual atomic fluctuation seems to be higher in case of FSH^24^(TAG) from dgFSH and FSH^15^(TAG) with a margin of 0.13 Å and 0.08 Å, respectively. Differences in the RMSF values of the protein for FSH^15^(TAG) and FSH^24^(TAG) from dgFSH are shown in Fig 8b. Substantial differences exist for residues: Ser17—Ile23, Val66-Phe72, Lys128—Arg132, Gly248, Asn382, Asp390, Gly394—Ser396, Leu411—Tyr414 and Thr426—Lys432.

#### 3.2.4 Flexibility of free and receptor-bound FSH

**Fig 9:**
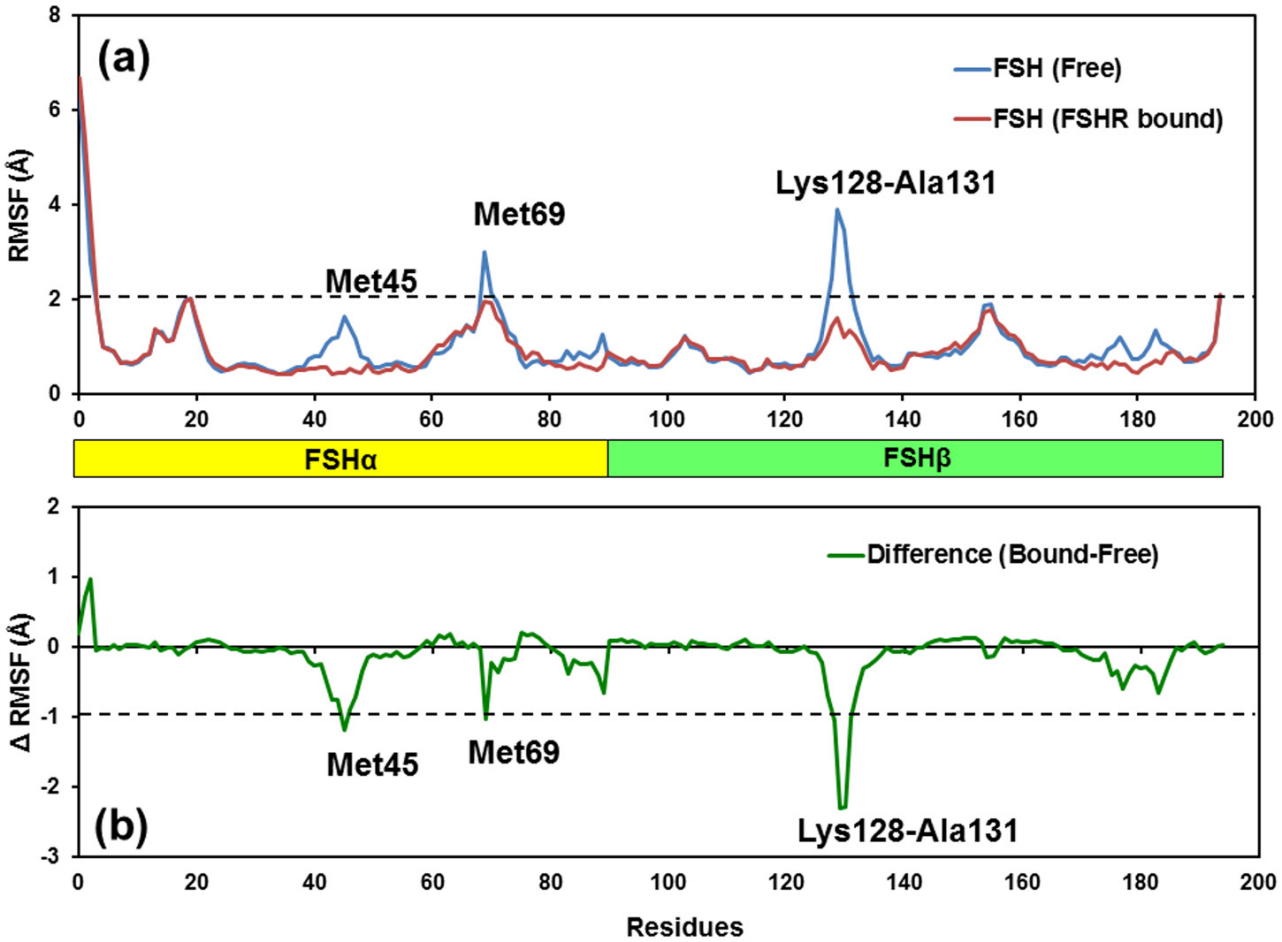
**(a)** Plot showing RMSF values of Cα atoms from MD simulations of free/unbound FSH and FSHr-bound models of FSH. RMSF changes were noted in two regions in the FSHα subunit centered on residues Met45 and Met69 and one region in the FSHβ subunit centered on residues Lys128-Ala131. Residues with RMSF changes of at least >2.0 Å are labeled inside the bars in each subunit. **(b)** Difference of RMSF values for FSHR-bound FSH and free-FSH models. The residues with absolute difference larger than 1.0 Å are labeled by one cutoff dashed black line.

Fig 9a compares RMSF values for free and FSHR-bound models of FSH and Fig 9b compare the difference between the RMSFs of free and FSH-bound models. Significant reductions in RMSF were noted in two regions in the FSHα subunit centered on residues Met45 and Met69 and one region in the FSHβ subunit centered on residues Lys128—Ala131. Reduced flexibility was noted for the region centered on αMet69 and a significant reduction in flexibility was associated with βLys128—Ala131. Both regions were located in the same region in FSH that formed the sulfo-Tyr335 binding pocket. Met45 was located in at the other end of the FSH molecule in αL2, which engages the receptor. Flexibility in two regions near the amino terminus of FSHα and in the C-terminal end of FSHβ was unaffected by binding to the FSHR.

### 3.3 Total binding free energies

**Fig 10:**
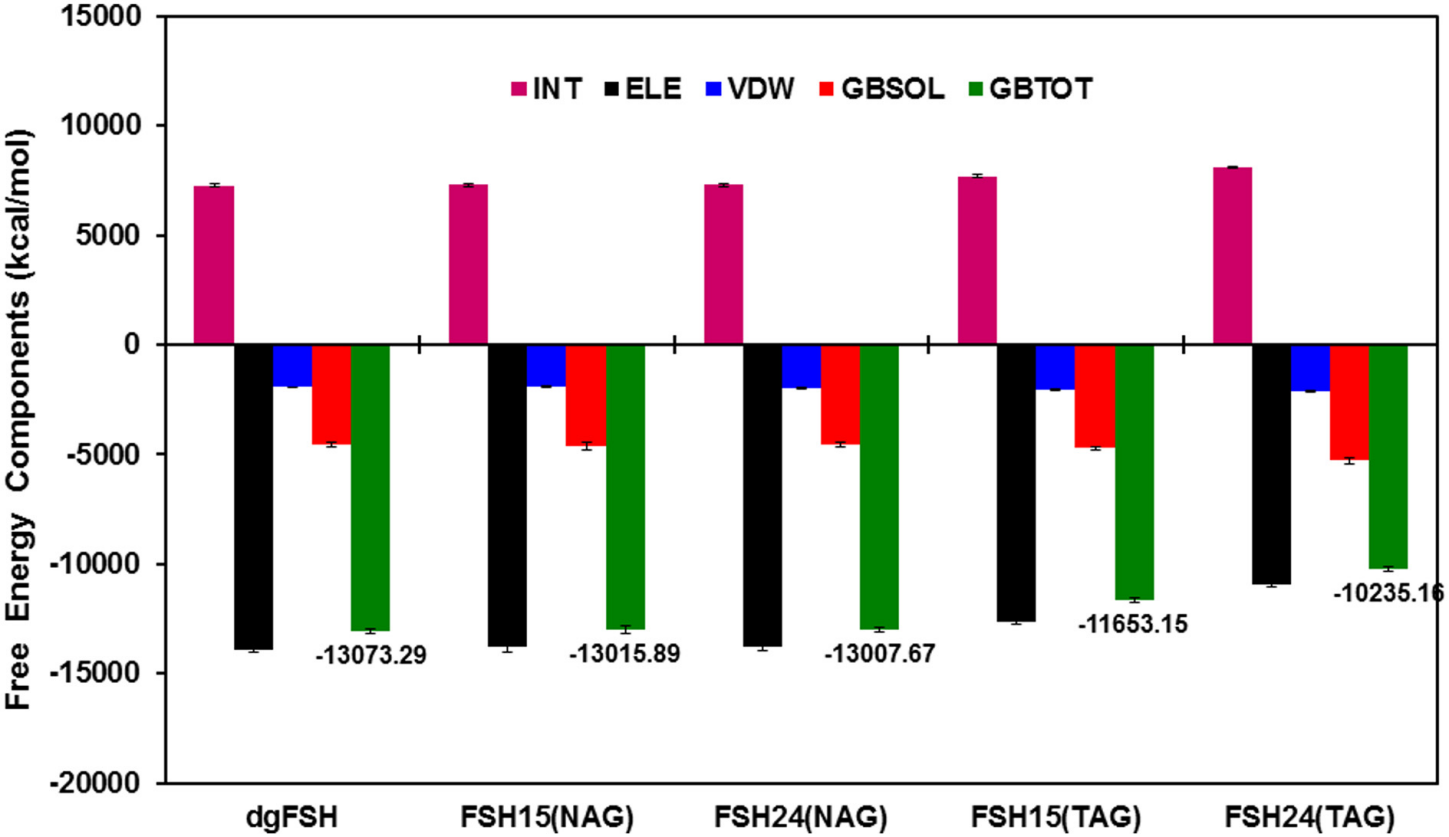
Energy components (kcal/mol) for the binding of FSH/FSHR in different systems like the dgFSH, FSH^15^(NAG), FSH^24^(NAG), FSH^15^(TAG), and FSH^24^(TAG): INT: Internal energies arising from bond, angle, and dihedral terms, ELE: Electrostatic energy in the gas phase; VDW: van der Waals energy; GBSOL: sum of polar and non-polar solvation energies; GBTOT: Total binding free energies. Error bars shown in black solid line specifies the difference in terms of standard deviations.

In order to assess the thermodynamic factors underlying differential FSHR binding affinity as a function of FSH glycosylation state, our GBSA calculations reveal that substantial differences arise in the FSH binding free energy for different complexes. Fig 10 and Table 1 reveals that the fully deglycosylated FSH binds with greatest affinity (GBTOT = total GBSA binding free energy) to the cellular FSH receptor. Smaller NAG glycans effect, if anything, only a minimal energetic perturbation to complex stability: the total binding energy for the FSH^15^ and FSH^24^ ligands exhibit no real statistical difference relative to the fully deglycosylated protein. With the caveat that differences in the GBSA free energy numbers are below the threshold for statistical significance, one may note that the appearance of a modest possible trend toward decreasing complex stability as a function of greater FSH glycosylation. This trend is substantially magnified for the larger TAG glycans where there is a large and significant decrease in free energy stabilization in comparing the deglycosylated and partially glycosylated states. Among the TAG isoforms, a similarly large difference in binding affinity is predicted in comparing partially- and fully-glycosylated FSH.

**Table 1:** FSH/FSHR binding free energies as computed from GBSA molecular dynamics studies. Values are in kcal/mol. Parenthetical values represent standard deviations in the free energies.

Energy Components	dgFSH	FSH^15^(NAG)	FSH^24^(NAG)	FSH^15^(TAG)	FSH^24^(TAG)
Internal Energy Δ*E*_int_	7274.46 (72.07)	7297.21 (68.66)	7307.17 (70.01)	7701.06 (62.26)	8118.63 (56.35)
Electrostatic Δ*E*_ele_	-13883.53 (117.07)	-13797.88 (203.03)	-13802.93 (133.97)	-12616.12 (99.75)	-10945.91 (130.25)
van der Waals Δ*E*_vdw_	-1932.3 (20.80)	-1913.37 (30.70)	-1962.31 (31.51)	-2040.92 (49.93)	-2123.45 (50.04)
Non-polar and Polar solvation Δ*G*_sol_ **(GBSOL)**	-4531.92 (95.29)	-4601.85 (171.04)	-4549.6 (111.06)	-4697.16 (80.48)	-5284.43 (119.37)
Free Energy Δ*G*_bind_ **(GBTOT)**	**-13073.29** (80.25)	**-13015.89** (72.16)	**-13007.67** (66.04)	**-11653.15** (77.85)	**-10235.16** (68.28)

The physicochemical origins of the glycoform-dependent variations in binding free energy are somewhat intricate. Two of the terms listed in Table 1 actually tend to favor increased binding as a function of increased FSH glycosylation: GBSOL (which accounts for complex entropy and desolvation effects) and van der Waals enthalpic contributions (which basically indicate that the more heavily glycosylated FSH isoforms have more favorable non-polar contacts than bare FSH). Conversely, and more dramatically, the covalent enthalpy (a measure of intra-monomer conformational strain) and electrostatic interaction components both show strong inverse correlations relative to increasing glycosylation.

### 3.4 Analysis of the salt-bridges

**Fig 11:**
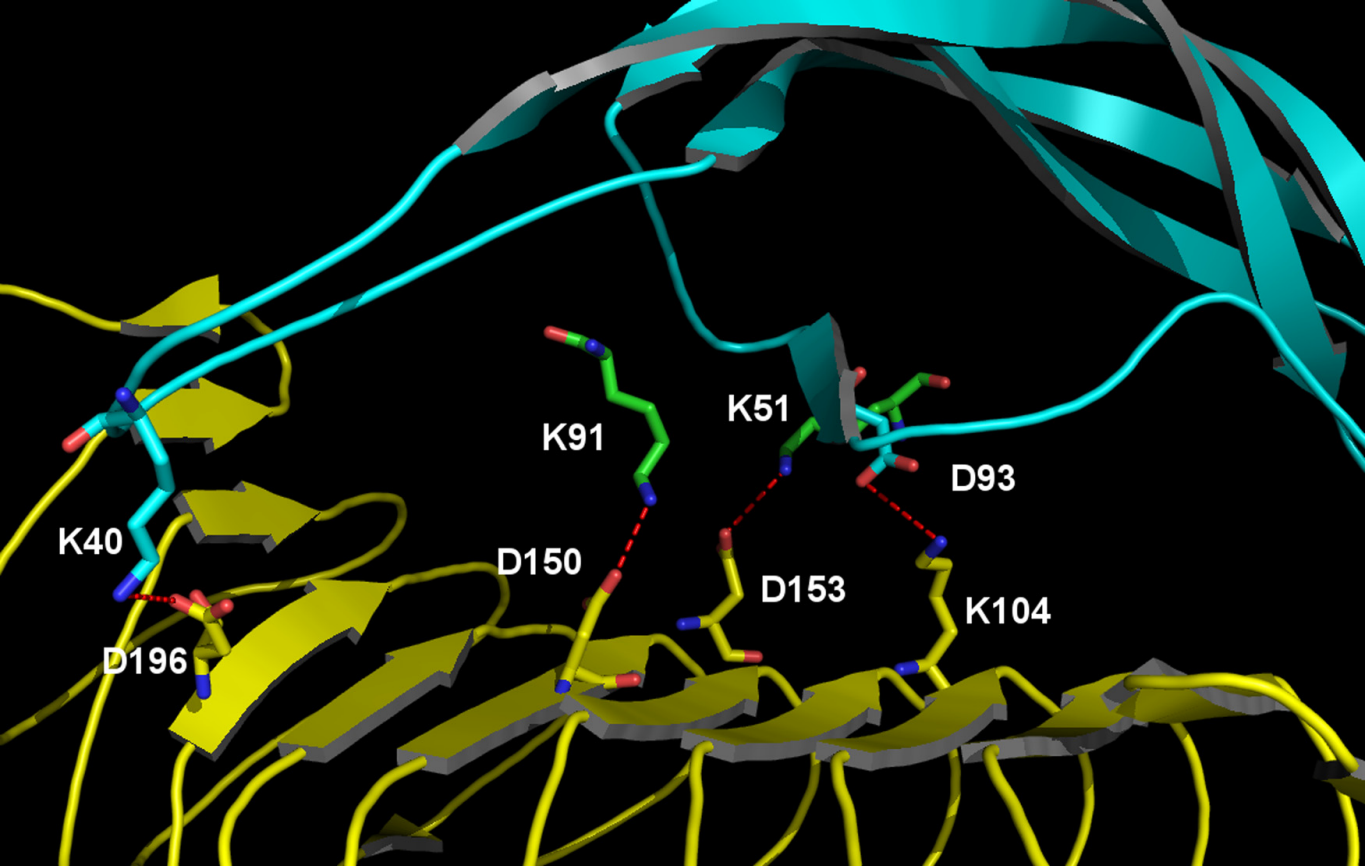
Key FSH/FSHR interface salt bridges underlying the electrostatic distinctions between FSH^15^(TAG) and FSH^24^(TAG). FSH is depicted as cyan ribbons with key residues shown as green/CPK-colored sticks, whereas FSHR is rendered via yellow ribbons and yellow/CPK-colored sticks.

Conformational strain within protein molecules is somewhat difficult to unambiguously characterize because it is frequently distributed across the molecule and can vary substantially in a dynamic fashion. The inter-monomer electrostatic coupling can be more readily interpreted, however. A key component of the FSH/FSHR interaction is mediated by a series of fairly strong salt bridges (two binding FSHα to FSHR and two between FSHβ and FSHR). These four key examples are depicted in Fig 11. As summarized in Table 2, the capacity of an FSH/FSHR complex to sustain each of these salt bridges varies significantly as a function of glycosylation state. In comparing fully deglycosylated FSH with FSH^15^(TAG) and FSH^24^(TAG) we find one salt bridge (emanating from Asp93 on FSHβ) to retain nearly invariant strength and persistence regardless of FSH glycosylation state, but the other three exhibit tangible variation. The coupling between Lys51 on FSHα and Asp153 on FSHR is modestly weaker and less persistent for FSH^15^(TAG) than is the case for FSH^24^(TAG), but more substantial salt bridge disruption is evident for FSH^24^(TAG), with significantly weaker and less persistent bonds originating from Lys91 on FSHα and from Lys40 on FSHβ.

**Table 2:** Median O-N distances for selected inter-protein salt bridges. Values are in Angstroms, averaged of molecular dynamics conformational sampling. Parenthetical values represent the fraction of time in which the O-N distance is less than 3.5 Å.

Salt Bridge	dgFSH	FSH^15^(TAG)	FSH^24^(TAG)
D93(FSH_β_)-K104(FSHR)	2.79 (99.8)	2.8 (100.0)	2.77 (99.8)
K51(FSH_α_)-D153(FSHR)	2.81 (99.7)	2.99 (97.1)	2.79 (99.5)
K91(FSH_α_)-D150(FSHR)	2.68 (100.0)	2.68 (100.0)	2.98 (90.6)
K40(FSH_β_)-D196(FSHR)	2.84 (96.5)	3.12 (77.9)	3.71 (61.9)

### 3.5 Solvent Accessible Surface Area (SASA) analysis

**Fig 12:**
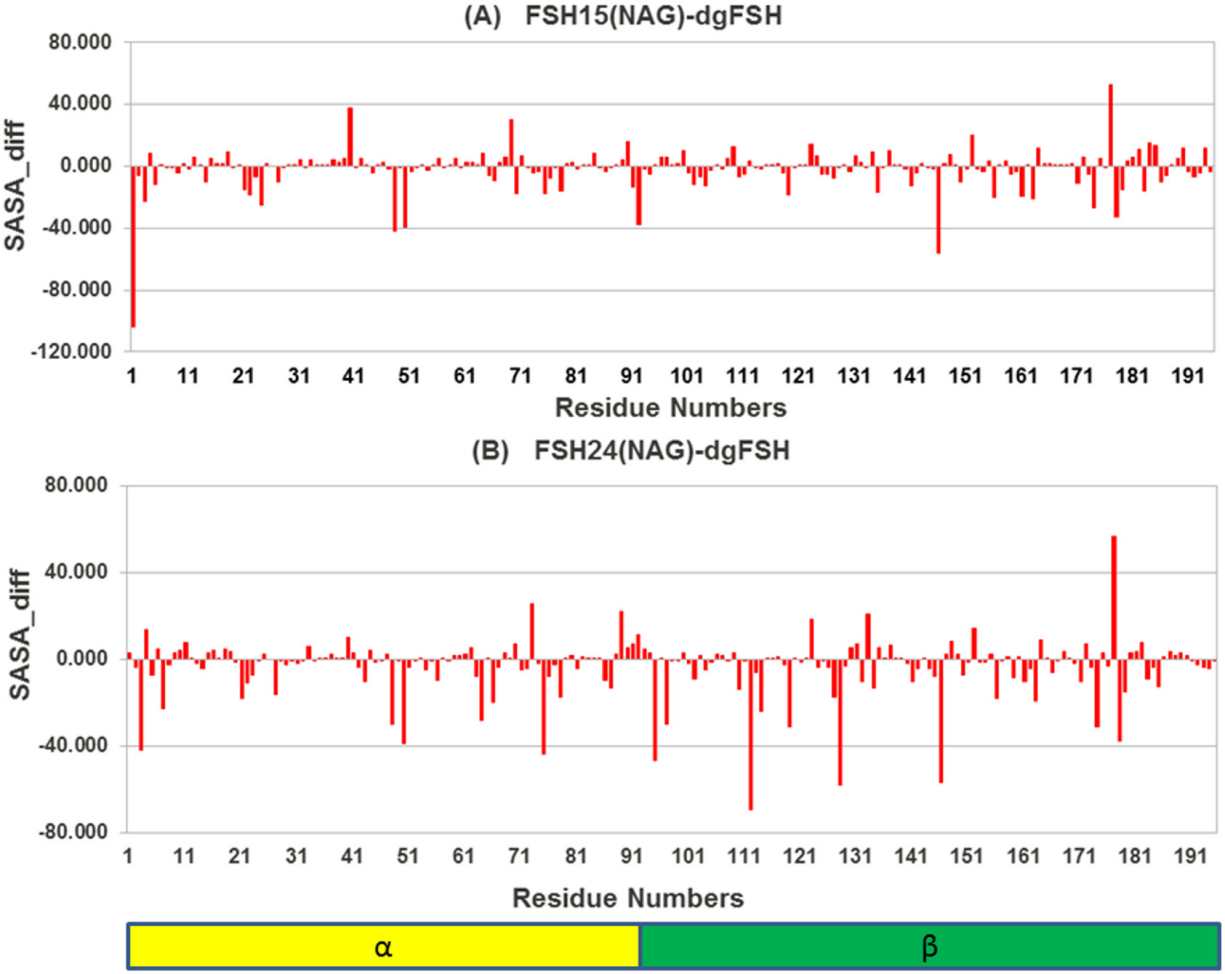
Difference in Solvent Accessible Surface Area (SASA) for FSH^15^(NAG) and FSH^24^(NAG) relative to de-glycosylated FSH.

**Fig 13:**
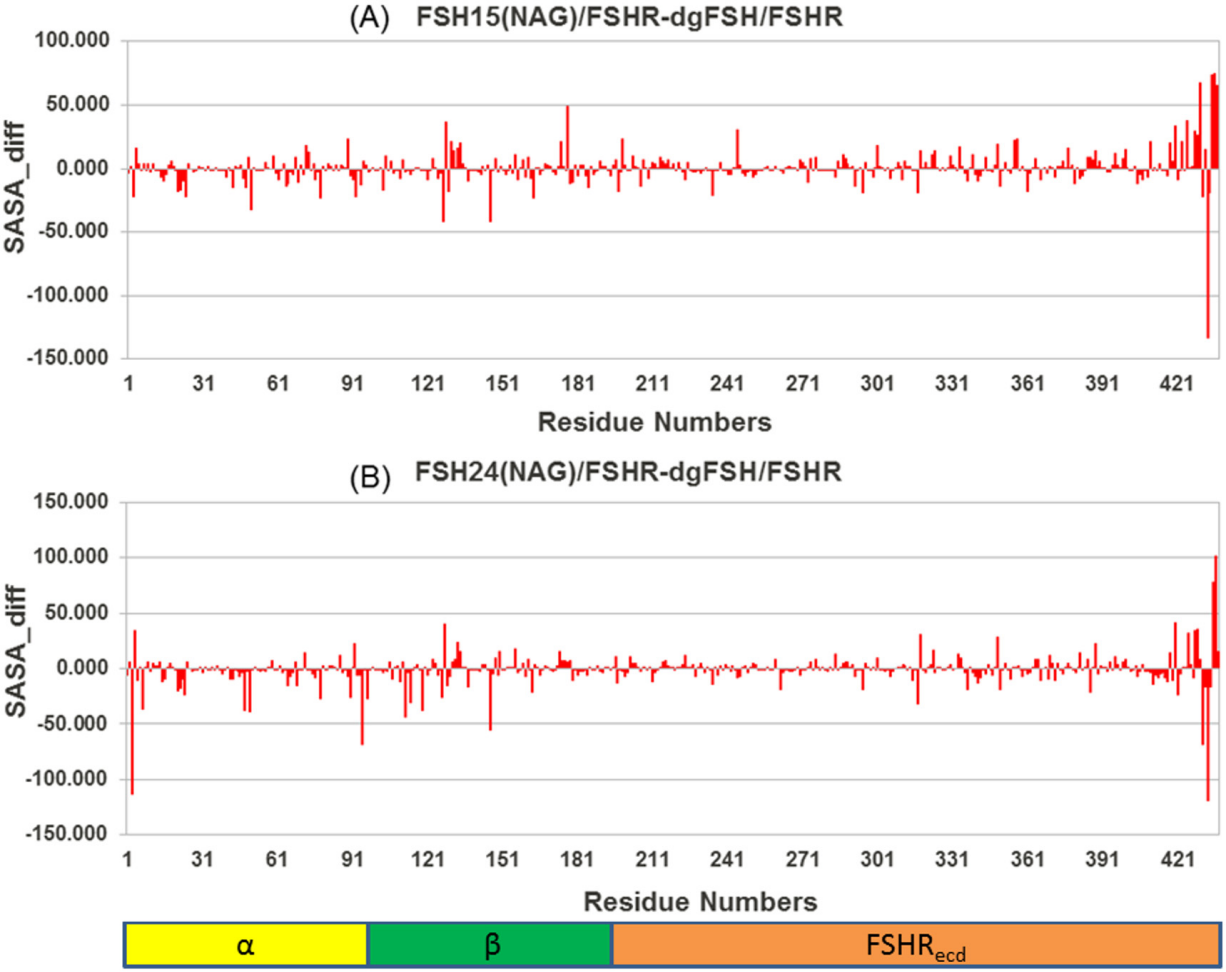
Difference in Solvent Accessible Surface Area (SASA) for FSH^15^ (NAG)-FSHR and FSH^24^(NAG)-FSHR from dgFSH.

**Fig 14:**
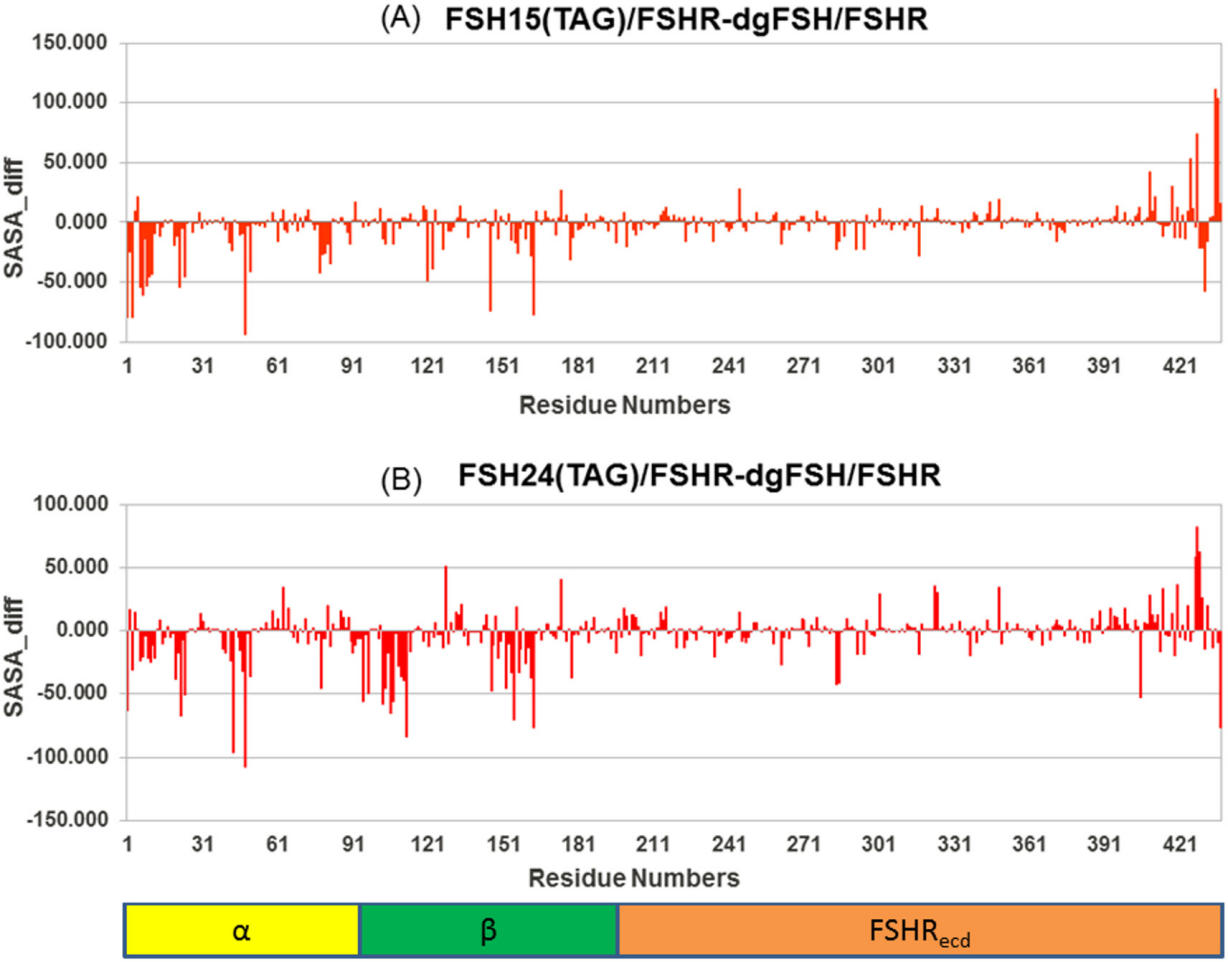
Difference in Solvent Accessible Surface Area (SASA) for FSH^15^ (TAG)-FSHR and FSH^24^(TAG)-FSHR from dgFSH.

The solvent accessible surface area (SASA) of a molecule is the area of the molecular surface that is exposed to the solvent. The free-energy of a protein due to the solvent is roughly proportional to the SASA of the molecule. The glycans reduce the SASA of the protein chain by a large amount as a result of steric hindrance by the glycan chains, especially the bigger ones, which may interfere in the complexation. The change in average SASA during the simulation (starting after the first 5 ns, when all the simulations are quite stable) on per residue basis was calculated for a variety of differences in FSH glycosylation state, and are plotted in Figs 12, 13 and 14. It can be seen that some of the important residues at the FSH-FSHR interface are clearly hindered by the presence of glycan chains.

#### 3.5.1 dgFSH—FSH^15^ and dgFSH—FSH^24^ differences

As shown in Fig 12, the differences of residues in either the FSH^15^ or FSH^24^ forms of the protein from dgFSH revealed few residues that contributed significantly. In order to identify the residues with a substantial contribution towards the SASA, we have categorized all residues using a cut–off of 40 Å contributions. Residues like Asp1, Gln48, Tyr146, and Ser177 showed differences for dgFSH—FSH^15^ (Fig 12a) and residues like Gln3, Glu75, Asn95, Asn112, Lys128, Tyr146 and Ser177 for the dgFSH—FSH^24^ comparison (Fig 12b). It was found that Tyr146 and Ser177 showed differences in both the FSH^15^ and FSH^24^ forms. Also their presence in the interface region enhanced the chance of their being important residues involved in the FSH-FSHR interaction.

#### 3.5.2 dgFSH—FSH^15^(NAG)-FSHR and dgFSH—FSH^24^(NAG)-FSHR differences

Fig 13 shows the differences for residues in dgFSH—FSH^15^(NAG) and dgFSH—FSH^24^(NAG). In order to identify the residues with a significant contribution towards the SASA, we categorized all residues using a cut–off of 40 Å contributions. Residues like Tyr127, Tyr146, Ser177, Leu430, Leu433 and Thr435, Leu436 and Glu437 showed a difference of more than 40 Å for dgFSH—FSH^15^(NAG) (Fig 13a) and residues such as, Gln3, Asn95, Asn112, Lys128, Tyr146 and Lys420, Lys431, Thr435, and Leu436 for the dgFSH—FSH^24^(NAG) comparison (Fig 13b).

#### 3.5.3 dgFSH—FSH^15^(TAG)-FSHR and dgFSH—FSH^24^(TAG)-FSHR differences

Fig 14 shows the residue-by-residue differences in solvent accessible area for dgFSH—FSH^15^(TAG) and dgFSH—FSH^24^(TAG) as well as highlighting residues contributing more than 40 Å for SASA. Residues like Asp1, Gln3, Pro6, Glu7, Thr9, Leu10, Gln11, Pro22, Leu24, Gln48, Asn50, Tyr121, Tyr146, Thr163, His409, Arg425, Tyr428, Lys431, Thr435, and Leu436 exhibited a difference of more than 40 Å for the dgFSH—FSH^15^(TAG) comparison (Fig 14a), while residues such as, Asp1, Pro22, Leu24, Lys43, Asp48, Asn78, Asn95, Thr97, Glu103, Glu104, Arg106, Phe107, Asn112, Tyr146, Pro152, Ala155, Thr163, Asn284, Asn285, Arg405, Thr427, Tyr428, Asn429, and Glu437 distinguished the dgFSH—FSH^24^(TAG) comparison (Fig 14b).

## Discussion

Evidence for the physiological existence of multiple FSH glycoforms within the same organism suggests that the biochemical function of FSH is glycoform-dependent. In other words, it is likely that FSH interaction profiles with its cognate cellular receptors will vary as a function of glycosylation state, thereby producing differential effects on related signaling pathways. This study has used MD simulations to examine the hypothesis [12] that hypo-glycosylated FSH glycoforms have the greatest functional efficacy in promoting reproductive function in young human females of child-bearing age. Given uncertainty as to the precise biochemical changes induced by deglycosylation at Asn7 vs. Asn24 on FSHβ, we have chosen in this preliminary study to simulate the FSH/FSHR binding profile for FSH^15^ which entails deglycosylation at both FSHβ sites, and compare this behavior relative to fully-glycosylated FSH^24^ and fully-deglycosylated, dgFSH. These simulations have been applied to the specific question of whether there is a kinetic or thermodynamic basis for predicting an enhanced binding profile for FSH^15^ (relative to FSH^24^) in its interactions with the high affinity gonadal FSH cellular receptor site.

FSH binding to its receptor involves three steps [17,20]. The first step consists of FSH engaging the high affinity binding site known to reside in the leucine-rich repeat region of the extracellular domain [7,16]. High affinity binding triggers the second step, a conformational shift in receptor-bound FSH, creating a binding site for a highly conserved sulfo-tyrosine residue that has been shown to be essential for glycoprotein hormone receptor activation [33]. The third step involves the FSH receptor sulfo-Tyr residue 335 (sTyr335) engaging the binding-induced site on FSH. This step has been proposed to remove the inhibitory influence of the unoccupied extracellular domain, permitting a shift from inactive to active conformation in the transmembrane domain, thereby activating the FSH receptor [17,20]. Because high affinity binding is necessary for subsequent steps, the studies reported herein are very relevant to a complete understanding of FSH-induced activation of the FSH receptor. While only the 1WXD crystal structure of FSH bound to the high affinity site of the FSH receptor extracellular domain was available when these studies were initiated, they remain relevant as all subsequent steps leading to FSH receptor activation are dependent on the initial high affinity interaction between the ligand and receptor. The subsequent crystal structure of FSH bound to the complete extracellular domain confirmed the interaction between FSH and the FSH receptor, as these interactions were the same in the 4AY9 structure. This latter structure revealed the significance of the conformational change attendant to high affinity FSH binding, which was not recognized in the earlier study [20].

The information collectively encompassed in Figs 4 and 5 clearly indicate that while different NAG-glycoforms of FSH do not appear to exhibit major conformational variations upon binding to FSHR, the FSH^15^(TAG) structure has an FSHR binding profile that is quite distinct to that which is observed for FSH^24^(TAG). Specifically, the FSH^15^(TAG) structure adapts much more quickly to forces exerted by FSHR than does FSH^24^(TAG). Furthermore, when spatially decomposing the structural adaptation, one finds that FSH relaxation in close proximity to the FSH/FSHR binding interface is substantially greater in the case of FSH^15^ than one finds for FSH^24^. This collectively appears to effectively corroborate experimental findings that FSH^18/21^ preparations associate significantly more rapidly with FSHR than does FSH^24^ [18].

Comparisons of the RMSD values for both FSH models observed in the FSH crystal structure [19], both FSH models in the FSH-FSHR hormone binding domain [16], and all three models found in the crystal structure of FSH and the FSHR extracellular domain [20] suggested a progressive loss of flexibility [17]. Loss of flexibility is consistent with dissociation studies in which ^125^I-FSH bound to the FSH receptor remains bound for up to 24 hr at 25°C in the absence of cold, competing FSH [34]. For the most part the regions of reduced flexibility were associated with the hairpin loop regions. In the present study, comparison of RMSF values indicated reductions in flexibility in three regions and no change in two other regions of the FSH molecule. Reduced flexibility for αMet69 and βLys128-Ala131 was associated with formation of the sTyr335 binding site. Conformational change associated with the βL2 loop was previously noted to push αL3 against αL1 [16]. The results reported herein are consistent with the concept that high affinity binding to the FSHR hormone binding region, which lacks sTyr335, creates a stable binding site for sTyr335 [20]. Reduced flexibility at αMet45 is probably due to its position in the middle of 5 residues buried in the FSH/FSHR interface [16].

Our calculations suggest that the functional differences between FSH^15^ and FSH^24^ are not strictly limited to associative kinetics. GBSA binding free energy calculations suggest that, while there is minimal variation in FSHR associative energy as a function of FSH glycoform, when the protein is adorned with small NAG glycans, a much greater energy variation is found when examining FSH decorated with large TAG glycans. Specifically, the free energy for FSH^15^ binding to the FSHR is predicted to be about 1418 kcal/mol more favorable than for FSH^24^. A fraction of this difference arises from greater intramolecular strain inherent in FSH^24^ while it is bound to FSHR, the bulk of the distinction is derived from differences in electrostatic affinity between FSH and FSHR. Most of that distinction can be distilled down to the behavior of two specific salt bridges: the binding of Lys91 on FSHα with Asp150 on FSHR and the coupling of Lys40 on FSHβ and Asp196 on FSHR. In both of these cases, the mean O-N ionic distance increases substantially for FSH^24^ binding to FSHR relative to what is predicted for FSH^15^, while the fraction of time the two ionic atoms remain within a moderately strong associative distance decreases significantly for FSH^24^. These observations provide clear evidence for conformational differences between FSH^15^ and FSH^24^ that selectively favor stable FSHR binding by the former relative to the latter.

It may be of interest to reflect on the behavior among the other GBSA terms. As mentioned above, when complexed with FSHR, the intra-molecular strain energy in FSH^24^(TAG) is higher than in FSH^15^(TAG). In addition to the natural thermodynamic penalty associated with a higher internal strain, it is likely FSH^24^(TAG) also experiences a net kinetic disadvantage due to a requirement, upon complexation, of having to travel further 'uphill' along the potential energy surface in order to achieve a stable complex with FSHR. Two terms in which the trends run in reverse to the above (i.e., FSH^24^(TAG) is favored relative to FSH^15^(TAG)) are van der Waals enthalpy and GBSOL (which collectively accounts for complexation entropy and desolvation enthalpy). The fact that FSH^15^(TAG) and FSH^24^(TAG) exhibit opposite trends among electrostatic and van der Waals enthalpies in associating with FSHR suggest that there may be a significant dielectric dependency influencing the relative FSH^15^(TAG) and FSH^24^(TAG) binding profiles, with low dielectric environments tending to favor FSH^15^(TAG) binding and a more polar environment favoring FSH^24^(TAG). The substantial difference in GBSOL may in part arise from a substantial solvation energy difference between the two FSH glycoforms, which again relates closely to the local dielectric environment. Since our modeling assumed a polar, aqueous environment which should be a fair (but unlikely perfect) approximation to the environment incumbent in the interstitial medium in close proximity to the high affinity gonadal (or other) FSH receptor site. Differences from a standard aqueous dielectric medium (ε = 80.4) should thus produce some degree of variation in the predicted binding free energies. Conversely, temperature variations may affect the relative entropy component within GBSOL and thus produce some environment-dependence in free energy predictions. However, given the relatively modest variations in GBSOL (relative to the covalent strain and electrostatic terms) we are confident that the simulations we have undertaken (and the assumed conditions thereof) are providing us with a reasonable and balanced deconvolution of the binding free energies.

## Conclusion

Ultimately, our evidence tends to point toward FSH^15^(TAG) having a more favorable FSHR binding profile than FSH^24^(TAG), both from a kinetic and from a thermodynamic perspective. Producing a relative weighting to these two factors to determine whether either clearly dominates the observed differences in binding behavior could be pursued through studies that explore the effects of temperature variation, but are beyond the scope of this preliminary study.
